# A Novel Surface Recovery Algorithm for Dual Wavelength White LED in Vertical Scanning Interferometry (VSI)

**DOI:** 10.3390/s20185225

**Published:** 2020-09-13

**Authors:** Linlin Zhu, Yuchu Dong, Zexiao Li, Xiaodong Zhang

**Affiliations:** State Key Laboratory of Precision Measuring Technology & Instruments, Centre of MicroNano Manufacturing Technology, Tianjin University, Tianjin 300072, China; l_linzhu@tju.edu.cn (L.Z.); 3014202004@tju.edu.cn (Y.D.); zexiaoli@tju.edu.cn (Z.L.)

**Keywords:** vertical scanning interferometry, dual wavelength white LED, recovery algorithm

## Abstract

The two peaks characteristic of yellow and blue light in the spectrum of dual-wavelength white light emitting diodes (LEDs) introduce distinctive features to the interference signal of white light scanning interferometry (WLSI). The distinctive features are defined as discontinuities, so that the fringe contrast function cannot be modeled as a single Gaussian function, and causes the interferogram to have uneven distribution of fringes of different orders in the scanning interferometer. This phenomenon leads to the low accuracy of the zero-order fringe position in the envelope calculation, which affects the repeatability and accuracy of the interferometry. This paper proposes a new surface recovery algorithm based on the Hilbert phase envelope and adjacent reference points calculation, which can effectively overcome the influence of the discontinuous signal of dual-wavelength LED white light interference on the three-dimensional reconstruction of WLSI measurements. The reliability of the algorithm is verified by experiments, and the measurement accuracy of LED WLSI system is evaluated.

## 1. Introduction

**Figure 1:**
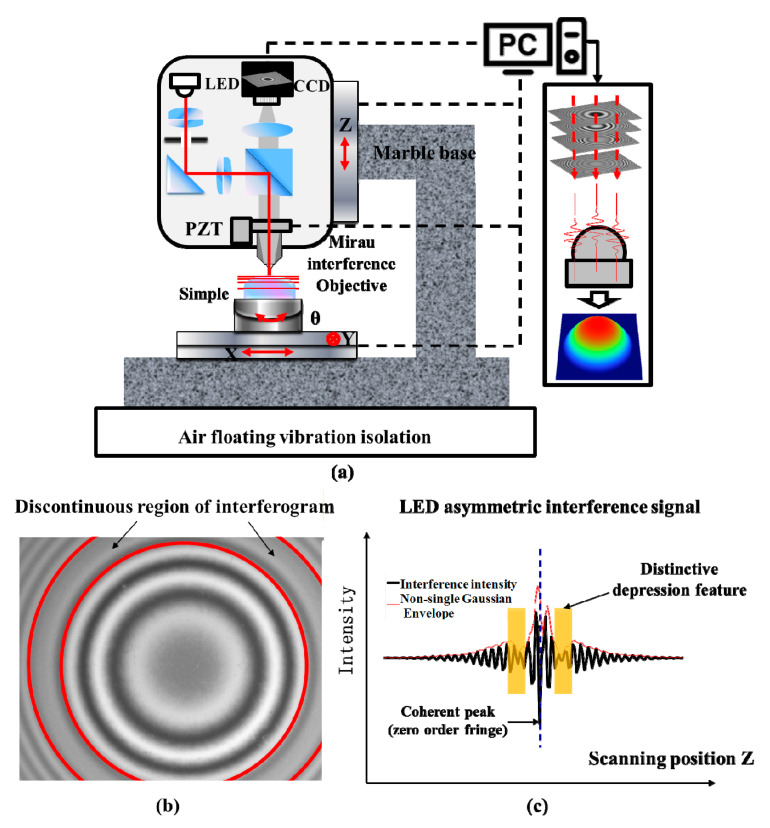
(**a**) The schematic of the white light scanning interferometry (WLSI) setup and interference data of dual wavelength white light emitting diodes (LEDs) (**b**) Interferogram; (**c**) Interference signal.

White-light scanning interferometry (WLSI) is an established optical method for surface profile measurement by analyzing a series of interference patterns of low coherence light with known optical path differences among them. A typical Mirau interferometer setup can be used for a WLSI system with a microscope arrangement, as shown in Figure 1a. In the imaging optical path with the broadband light source, a piezoelectric transducer (PZT) is used to vertically move the Mirau interference objective lens in the nanometer range to change the optical path difference between the sample and the beam splitter, and generate a series of moving interference fringes. The spatial distance between the surface measurement point and the equal optical path point can be obtained by collecting the interference fringes with charge coupled device (CCD) and calculating the position of the signal coherence peak, so as to realize the height recovery of the measurement surface with nanometer resolution [1,2]. Generally, the brightness of the light source in WLSI can easily affect the contrast of interference fringes, and its spectral width is related to the area where interference occurs. The wider the spectrum, the more information the measurement contains [3]. For decades, LEDs or tungsten–halogen lamps have provided the low-coherence light and, as phosphor-based white LED promises greater power, longer lifetime, low heat dissipation and compactness, it is replacing the conventional light source in WLSI [4]. However, white LEDs mainly synthesize white light by exciting yellow phosphors on blue or near ultraviolet LED chips, and its spectrum has dual wavelength characteristics of yellow and blue. As shown in Figure 1b,c, the distinctive feature is defined as the discontinuities which make the fringe contrast function unable to be modeled as single Gaussian function [5,6]. The LED WLSI interference light intensity signal is affected by the double peak of the light source spectrum, which leads to the asymmetric distribution of interference signal, and the interference signal depression position has the phenomenon of jumping at different detection heights. This brings a great challenge to some coherent peak extraction algorithms which require that the central wavelength of white light source is stable or the interference signal is symmetrical. The effects of the distinctive feature to the reconstructed height profile vary depending on the working principle of the reconstruction algorithm, and it has been shown that the measurement repeatability will degrade and produce poor surface reconstruction quality if the spectral effects are not treated properly [7].

At present, LED WLSI is still based on the existing conventional interference signal reconstruction method to obtain the morphology of the measured samples, which can be divided into two categories: the time domain modulation algorithm using interference signals or its envelope’s amplitude information [8,9] and frequency domain algorithm based on the signal’s spectrum phase analysis [10,11]. Among them, the time-domain modulation algorithm is used to find the position of maximum intensity (i.e. zero optical path difference) in the signal by extracting its envelope, and then determining the position of the signal’s coherent peak and restoring the surface topography. The second method is to extract the phase information of the white light interference signal through the phase shift algorithm or Fourier transform, and combine the envelope centroid algorithm to unwrap the wrapped phase to reconstruct the surface. However, the spectrum of the dual-wavelength white LED destroys the establishment conditions of the traditional interference signal envelope algorithm, and does not meet the assumptions adopted by each reconstruction algorithm. Due to the asymmetric interference signal characteristics and the problem of phase mutation, LED WLSI often has poor reconstruction quality. To solve this problem, Wee Keat Chong [5,12,13] proposed a local Gaussian fitting method to reduce the impact of LED dual-wavelength, and then proposed to perform band-pass filtering on the two peaks after Fourier transform of the LED spectrum, and then obtain the phase information for each filter window. Finally, they found and calculated phase-crossing points to locate the zero phase. The study was valuable and instructive. It achieved high-precision surface reconstruction to a certain extent and reduced the adverse effects of white LED characteristics. However, this algorithm may have multiple solutions when the measured element is a cyclic structure. Therefore, it is necessary to improve and enhance various kinds of complex and smooth surface to be measured. Jorez [14] studied the effect of the LED light source spectrum drift on the phase shift method. It is proposed that a correction factor can be calculated to compensate for the asymmetrical spectral shape to ensure the measurement accuracy of the sample contour. Unfortunately, the study of Kalle Hanhijärvi [15] demonstrated that the uncertainty of the phase shift method decreases with the change of the light source spectrum. Experiments verify that the uncertainty of the five-sample-adaptive (FSA) envelope method [16] is basically not affected by the change of the light source spectrum, but in terms of calculation effect, the phenomenon of rough surface and low reconstruction quality still exists. 

In order to solve the influence of the dual-wavelength characteristics of white LED on interference reconstruction, we proposed a new recovery algorithm with good robustness and high reliability based on actual measurement experience to avoid false detection of coherent peak positions. The algorithm obtains the relative height of the initial solution of each surface point based on the Hilbert phase envelope. Then, the reference height of the adjacent phase reference point is calculated, and the difference between the initial solution height and the reference height is compared to optimize the surface reconstruction result. This algorithm effectively decreases the negative impact caused by double peaks characteristic of the light source spectrum in the surface reconstruction for the LED WLSI, and solve the problems of envelope peak extraction inaccuracy and the difficulty of solving the phase mutation in the traditional algorithm to a certain extent. Finally, the reliability of our algorithm is verified by experiments.

## 2. Influence Analysis of Dual-Wavelength White LED Spectrum on Interference Signal

### 2.1. Simulation of Interference Intensity Signal of LED

The white light interference signal can be expressed by Equation (1) [17]. The interference signal can be described as the sum of the interference components *g*(*β*, *k*, *ζ*) at each wave number *k*, which is modulated by the spectrum intensity distribution function *V*(*k*) of the light source and the function *βU*(*β*) related to the exit angle *β* of the measurement system.

(1) $$I\left(\zeta \right)=\overset{\infty }{\underset{0}{\int }}\overset{1}{\underset{0}{\int }}g\left(\beta ,k,\zeta \right)U\left(\beta \right)V\left(k\right)\beta d\beta dk$$

where *ζ* is the position of each measurement step. The empirical formula of Equation (1) is generally used to calculate the light intensity information of white LED interference signal [18], namely

(2) $$I\left(z\right)=C\overset{\infty }{\underset{0}{\int }}\overset{\theta_{0}}{\underset{0}{\int }}\left\{k^{2}cos\left[2k\left(z-z_{0}\right)cos\theta +\Phi \right]sin\theta cos\theta \right\}V\left(k\right)dkd\theta$$

where *C* is a light intensity related constant, *z*_0_ is the height of the measured surface, *z* is the measurement position of the system, *θ*_0_ is related to the NA value of the interference objective lens (NA = *nsinθ*_0_, where *n* is the refractive index of the working environment medium of the objective lens), represents the phase deviation, and *V*(*k*) represents the spectrum intensity of the light source.

The simulation process assumes that there is no phase deviation *Φ* = 0. Let *C* = 1, the system objective NA = 0.4, the objective works in the air, and the refractive index *n* = 1. The simplified Equation (3) is obtained by simplifying *γ* = *cosθ*_0_ in Equation (2), and the simulated interference light intensity signal can be obtained by integral calculation.

(3) $$\begin{array}{l} I\left(z\right)=C\overset{\infty }{\underset{0}{\int }}-\frac{V\left(k\right)}{4z^{2}}[2kz\gamma \sin\left(2kz\gamma +\Phi \right)-2kz\sin\left(2kz+\Phi \right)\\ \;+cos\left(2kz\gamma +\Phi \right)-cos\left(2kz+\Phi \right)]dk \end{array}$$

**Figure 2:**
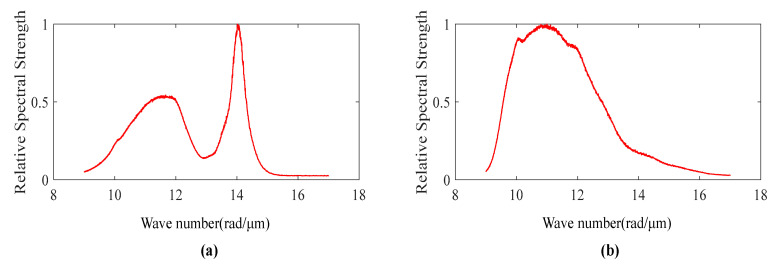
(**a**) Spectrum intensity distribution of a white LED light source; (**b**) Spectrum intensity distribution of a halogen light source.

**Figure 3:**
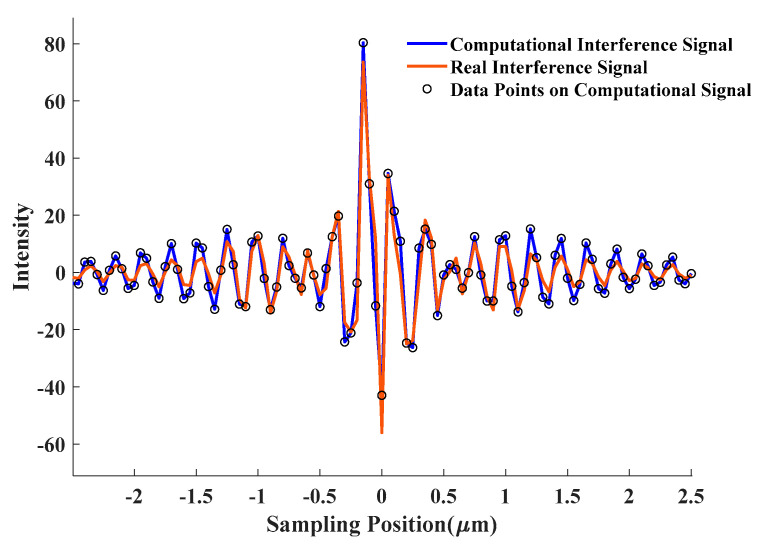
Comparison between the interference signal calculated by simulation and the interference signal measured.

Using a spectrometer to measure the spectral intensity distribution of the Cree LED light source and Thorlabs halogen lamp light source that are often used in interferometers. Figure 2a,b show the spectral intensity measurement results of the LED light source and the halogen lamp light source, respectively. There are two peaks in the LED spectrum intensity distribution due to the physical properties of the light source, while the spectrum distribution of the halogen lamp is close to a single Gaussian peak, and there is only one main peak in the spectrum. Then, the spectrum distribution of LED light source is used to simulate the white light interference signal through formula (3), and the result is compared with the interference signal obtained in the actual measurement process, as shown in Figure 3. The two signals are similar in form, which shows the correctness of the signal simulation method and can reflect the nature of the interference signal of the system.

**Figure 4:**
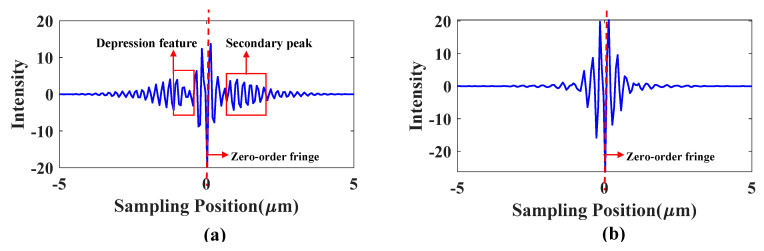
(**a**) Simulated white light interference signal of LED; (**b**) Simulated white light interference signal of halogen lamp.

The spectrum intensity distribution of LED light source and halogen light source are respectively used to generate interference signals for vertical scanning interferometry (VSI) measurement of a plane at a sampling interval of 50 nm through simulation. It can be seen in Figure 4 that both interference signals have high zero-order fringe visibility, but there exists difference between the halogen light interference signal with a single Gaussian peak white light spectrum and the LED interference signal. The white light interference signals of LED exhibit depression at the position about 0.6 μm away from the zero-order fringe, and there are secondary peaks at the position from 0.6 to 3.0 μm away from the zero-order fringe. This feature becomes a typical feature of the white LED interference signal, which is the key point of this paper.

### 2.2. Influence of Envelope Method on Zero-Order Fringe Positioning

**Figure 5:**
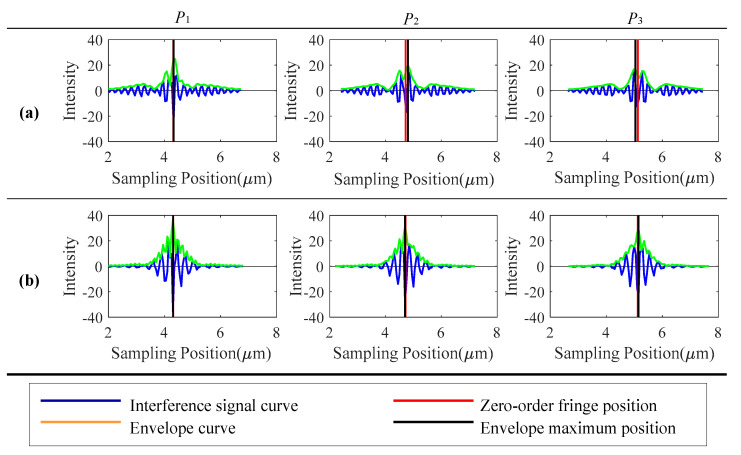
Fast Fourier transform (FFT) envelope zero-order fringe positioning results: (**a**) white LED light source (**b**) halogen light source.

**Figure 6:**
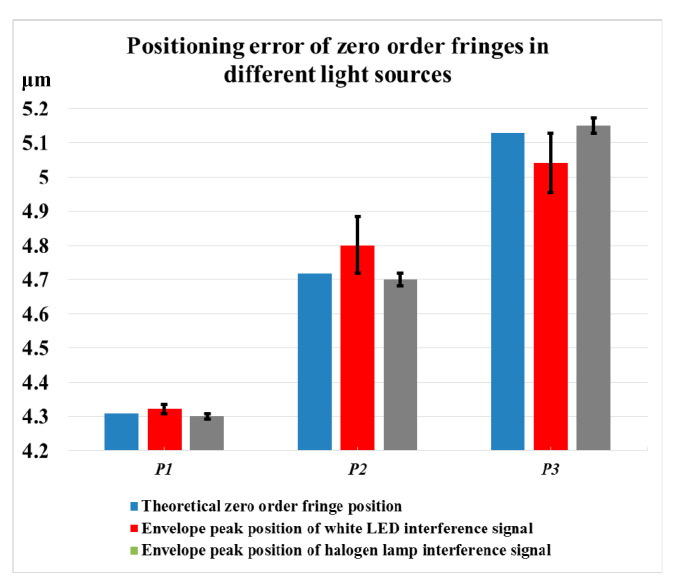
Envelope peak error of white LED.

The influence of the depression and the secondary peak of the white LED interference signal on the extraction of the position of the zero-order fringes in the WLSI is simulated and analyzed. Based on the interference signal simulation method introduced in Section 2.1, the simulated experiment is to measure an inclined plane. In the experiment, three different measurement heights (P_1_, P_2_ and P_3_) are randomly selected at equal intervals, and the interference signal envelope peak positions of white LED and halogen light source are calculated, respectively, based on the traditional fast Fourier transform (FFT) envelope algorithm [19]. The simulation results are shown in Figure 5. It can be seen from the figure that the envelope curve of white LED interference signal is seriously affected by the depression and secondary peak, which results in the phenomenon of poor symmetry of envelope curve and multiple envelope peaks. The phenomenon is more obvious when the depression is closer to the zero-order fringe. At different heights, there is a jump change in the position of the main peak of the envelope, and the deviation from the center position of the theoretical zero-order fringe is large. Meanwhile, the curve of the interference signal of the halogen lamp has good symmetry, with an outstanding single-peak characteristic, and the deviation of the envelope main peak position from the zero-order fringe center position is small. The deviation of the envelope main peak position from the actual center position of the zero-order fringe will influence the positioning accuracy of the zero-order fringe position in the algorithm. As shown in Figure 6, the difference between the zero-order fringe center position and the actual position of the two light source interference signals is calculated by the FFT envelope algorithm. It is obvious that the zero-order fringe center position deviation calculated for white LED interference signal is large.

Considering that there is a direct relationship between the positioning accuracy of the envelope peak of the algorithm, a single reconstruction algorithm cannot fully reflect the influence of white LEDs on the positioning accuracy of the algorithm. Therefore, a variety of traditional reconstruction algorithms are used to solve the interference signals of two kinds of light sources at different heights on an inclined plane for further verification. In this paper, Centroid [20], FFT [19], and Stoilov white light phase shifting interferometry (WLPSI) [21] are used to calculate 40 groups of simulated white LED and halogen light source interference signals by measuring the same inclined plane at different heights with equal intervals based on simulation. The deviation between the calculation result of the center position of the zero-order fringe of the interference signal and the actual center position of the zero-order fringe at each height is obtained. The calculation results are shown in Table 1. Different reconstruction algorithms all have envelope peak deviation. The calculation accuracy and repeatability of the centroid method and FFT algorithm are good, and the positioning deviation of the phase-shift method is the largest. It can be seen from the comparative result of the two light sources that the positioning deviation of the white LED interference signal’s zero-order fringe is obviously larger than that of the halogen lamp using the same algorithm. A total of 40 groups of interference signals obtained by simulation are in ideal conditions, and the zero-order fringes positioning deviation of these algorithms is small relatively. However, in the case of actual measurement process, the positioning deviation will be more obvious, and the final measurement results are more unreasonable.

The simulation experiment results indicate that the dual-wavelength characteristic of the white LED light source does affect the resolution accuracy of its interference signal, leading to a decrease in measurement accuracy. Therefore, we need to mend the reconstruction algorithm of the white light interference signal, overcoming the adverse effects of the depression and secondary peak in the white light interference signal of the LED light source, and finally improve the measurement accuracy of the LED WLSI.

## 3. Surface Reconstruction Algorithm Proposed

**Figure 7:**
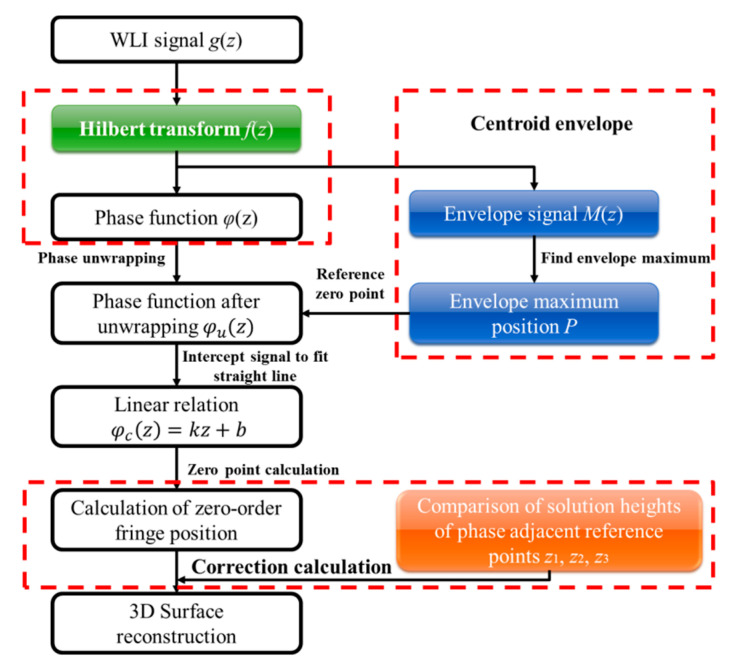
Flowchart of the proposed algorithm.

The flow chart of VSI interference signal reconstruction algorithm proposed in this paper is shown in Figure 7. In the first step, the Hilbert transform is applied to the interference signal. The Hilbert transform is a transform in the time domain. Compared with the Fourier transform, the calculation process of the Hilbert transforms and its inverse transform have a faster speed [22,23]. After preprocessing the interference signal, the phase of the interference signal is calculated using the 90° phase shift characteristic of Hilbert transform to obtain the wrapped phase [24,25]. The second step is to calculate the signal’s envelope and search the center position of zero-order fringe based on the centroid method to provide initial values for phase unwrapping. In the third step, a straight line is fitted to the unwrapped phase to obtain the minus one order, zero-order and one-order fringes’ center positions *z*_1_, *z*_2_, and *z*_3_. After completing every data point’s signal reconstruction, then the height reconstruction result of every data point will be compared with that of its adjacent reference points to correct its height using the correction algorithm illustrated in detail later. The surface height is reconstructed and modulated, and finally the 3D surface morphology is restored.

We set the interference signal obtained by measurement as *g*(*z*). The interference signal *g*(*z*) is transformed by Hilbert transform, and the transformation result is *f*(*z*). The frequency spectrum of *g*(*z*) is *G*(*k*), and the frequency spectrum of *f*(*z*) is *F*(*k*). Then the negative frequency components of the phase spectrum are shifted by +90°, and the positive frequency components of the phase spectrum are shifted by −90°. The Hilbert transform relationship is Equation (4).

(4) F(k)=−i×sgn(k)×G(k)

where *sgn*(*k*) is a symbolic function, *g*(*z*) and *f*(*z*) are used to calculate the envelope *M*(*z*) and phase function *φ(z*) of the interference signal through Equations (5) and (6).

(5) $$M\left(z\right)=\sqrt{g^{2}\left(z\right)+f^{2}\left(z\right)}$$

(6) $$\varphi \left(z\right)=\mathrm{atan}\left(\frac{f\left(z\right)}{g\left(z\right)}\right)$$

Then, the centroid method is used to calculate the position *P* of maximum value of the envelope *M*(*z*). The formula of centroid method is shown in Equation (7).

(7) $$P=\frac{\sum {\left(M\left(z\right)\right)}^{2}\times z}{\sum {\left(M\left(z\right)\right)}^{2}}$$

**Figure 8:**
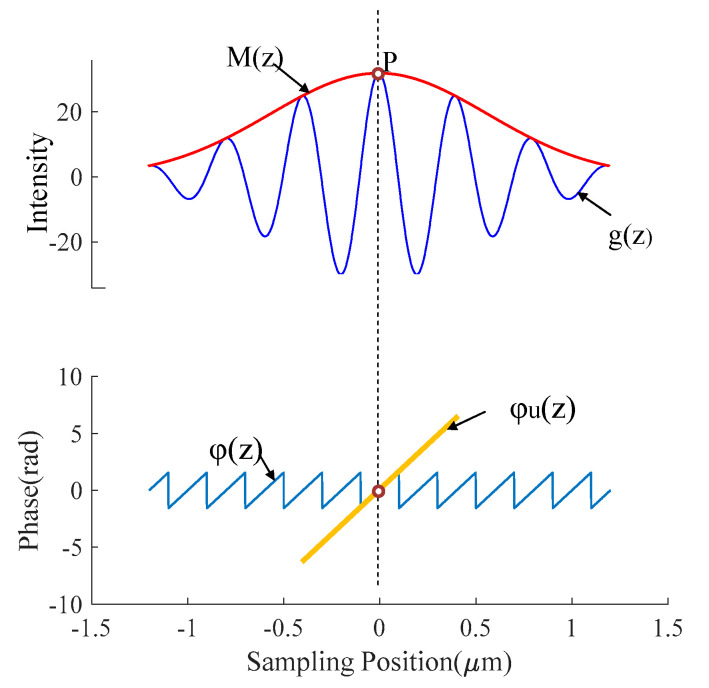
The envelope and phase curve of the vertical scanning interferometry (VSI) interference signal.

As shown in Figure 8, the zero point of the wrapped phase function *φ*(*z*) of the white light interference signal is located on the maximum value position P of the signal envelope *M*(*z*). So, *P* is regarded as the reference zero point used to unwrap *φ*(*z*), and the unwrapped phase function *φ_u_*(*z*) after unwrapping is obtained. Taking *P* as the center, *φ_u_*(*z*) is intercepted with *N* as the radius through Equation (8).

(8) $$\varphi_{c}\left(z\right)=\varphi_{u}\left(z\right)\times \left(\varepsilon \left(P-N\right)-\varepsilon \left(P+N\right)\right)$$

where *ε*(*P* – *N*) and *ε*(*P* + *N*) are step functions. *N* = (*λ*_0_/2/*Δt*) × 5, *λ*_0_ is the central wavelength of the white LED, *Δt* is the VSI step. In an ideal situation, the phase value at the center of the zero-order fringe (position *P*) of WLSI is zero. The least square method is used to fit *φ_c_*(*z*) in a straight line to obtain *φ_c_*(*z*) = *kz* + *b*. The position of the zero-order fringe is *–b/k* calculated by line fitting, and then the actual height of the measured surface corresponding to the zero-order fringe is calculated according to the phase shift of each step.

**Figure 9:**
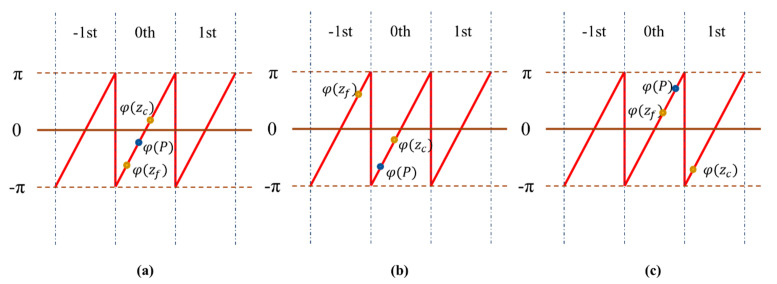
Schematic diagram of the phase deviation of the envelope peak: (**a**) *φ*(*z_c_*), *φ*(*z_f_*) and *φ*(*P*) in the same period; (**b**) and (**c**) *φ*(*z_c_*), *φ*(*z_f_*) and*φ*(*P*) in different periods.

However, in the actual system measurement process, due to the influence of the spectrum characteristics of the white LED light source, the envelope curve of the interference signal at different surface heights will change greatly, which affects the accuracy of the reconstruction algorithm. This paper improves the algorithm for the problems existing in the LED WLSI. In the proposed algorithm, it is considered that the zero-order fringe position of the interference signal envelope obtained by the centroid method is relatively accurate. There will be no large deviation of the zero-order fringe positioning and the positioning deviation will not exceed the position of –1 and +1 order fringes. After the center position P is obtained by the centroid method, two phase values of two integer position points *z_f_* and *z_c_* close to *P* are taken from the wrapped phase function *φ*(*z*) to determine whether the phase values of the two points are in the same [–π, π] period. If it is in the same period as shown in Figure 9a, this paper considers that the surface calculation result of this point will be more accurate, and this point will be recorded as a stable point. If it is not in the same period as shown in Figure 9b,c, the reconstruction result of this point will have a greater probability of having large deviation, and this point is recorded as an unstable point. The classification of points will be used as the determinant for the selection of starting point in the subsequent surface correction algorithm.

At the same time, the proposed algorithm calculates and records the values of three points *z*_1_, *z*_2_, and *z*_3_ on the *φ_c_*(*z*) line, and the three points, respectively, satisfy the condition of Equation (9)

(9) $$\{\begin{array}{c} kz_{1}+b=-2\pi \\ kz_{2}+b=0\\ kz_{3}+b=2\pi \end{array}$$

The three points in the formula correspond to the positions of the −1, 0 and +1 fringes’ center of the interference signal, and –2π and 2π are the phase values of the −1 and +1 fringes’ center after unwrapping. *z*_2_ will be used as the initial value of the surface height reconstruction result *z*_1_ and *z*_3_ will be used as the reference heights for reconstruction, and will participate in the surface correction.

**Figure 10:**
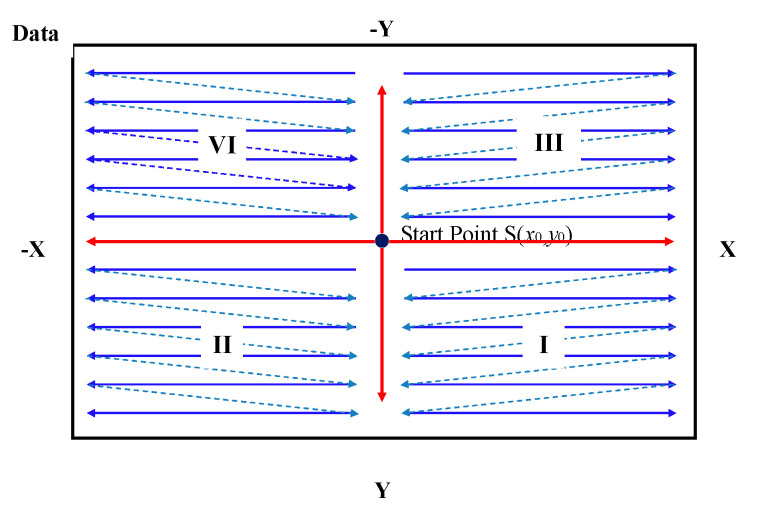
Schematic of Scan-Line path design strategy.

After completing the calculation of the interference signal of all pixels and obtaining the initial reconstruction result of surface, we proposed a Scan-Line correction algorithm to adjust the initial result to improve the surface reconstruction accuracy of the measured freeform surface, and reduce the adverse effect of the white LED light source on the surface reconstruction. The algorithm first finds a suitable point *S*(*x*_0_,*y*_0_) near the coordinate midpoint of the surface data as the starting point of the algorithm, and the starting point needs to be a stable point. After the starting point is determined, take the starting point as the center and scan along the *X*-axis positive direction, *X*-axis negative direction, *Y*-axis positive direction, and *Y*-axis negative direction to adjust the surface profile on the two axes. The algorithm scan path is shown in Figure 10. For continuous freeform surface measurement, according to the surface characteristics of freeform surface, it can be known that there does not exist sharp step change between two adjacent points, so a reasonable threshold can be set according to the actual measurement experience (300 nm is set in the algorithm in this paper). If the absolute value of the height difference between two adjacent points is greater than the threshold (the initial *z_2_* phase solution surface height deviation is large), it indicates that there exists large deviation with the reconstruction result of this point and needs to be adjusted. Compare the reference heights *z*_1_ and *z*_3_ of this point with the reconstruction height of its previous point and select the height value closest to the height of previous point between reference heights instead of this point’s initial height value *z*_2_, to correct the surface reconstruction result.

Here, the scanning correction process in the *X*-axis positive direction is taken as an example. The scanning point is set as (*x*,*y*_0_), and its former point (*x*–1,*y*_0_) is taken as the reference point, F(*x*, *y*) is the measured surface. If the absolute height difference |*F* (*x*, *y*_0_)–*F* (*x*–1, *y*_0_) | between two points is greater than the threshold, then the comparison of Equation (10) is performed and the height this point is corrected.

(10) $$F(x,y_{0})=\left\{ \begin{array}{l} z_{1}(x,y_{0})\left|z_{1}(x,y_{0})-F(x-1,y_{0})\right|<\left|z_{3}(x,y_{0})-F(x-1,y_{0})\right|\\ z_{3}(x,y_{0})\left|z_{1}(x,y_{0})-F(x-1,y_{0})\right|<\left|z_{3}(x,y_{0})-F(x-1,y_{0})\right| \end{array}\right.$$

After finishing the correction of the surface on the *X*-axis and *Y*-axis with the starting point as the center, the data are divided into four parts according to the *X*-axis and *Y*-axis. According to the scan line path design strategies: *X*-axis direction first and from the center to the edge, the algorithm generates the path to traverse all data points. According to the surface correction judgment shown in Equation (10), the initial reconstruction surface is corrected by traversing each data point. In Figure 10, the data is divided into four areas, I, II, III and IV, with the starting point as the center. In each region, the region I algorithm corrects the initial surface along the scanning path from left to right, from top to bottom; in region II, it follows the scan path from right to left and top to bottom; and region III follows from left to right and bottom to top; lastly, region IV follows the scan surface path from right to left, and from bottom to top. The algorithm can complete the correction of the surface by only traversing all points one time, which ensures the calculation speed. At the same time, the data in the four regions can be calculated simultaneously by multiple threads without interfering with each other, which can further improve the operation speed of the algorithm.

## 4. Feasibility Simulation 

In order to verify the accuracy of the algorithm proposed in this paper, based on the measured spectrum intensity distribution of the LED light source, 50 sets of white light interference signals on the upper and lower surfaces of the 4 μm height step are generated by the simulation. The sampling interval of the interference signals is 50 nm, and Gaussian white noise (zero mean, variance of 0.5) is added to the simulation measurement signals. The centroid, FFT envelope, Stoilov WLPSI method and the algorithm proposed in this paper, four different VSI interference signal solving methods are used to calculate these 50 sets of signals, and the accuracy and repeatability of the calculation between the algorithms are compared.

**Figure 11:**
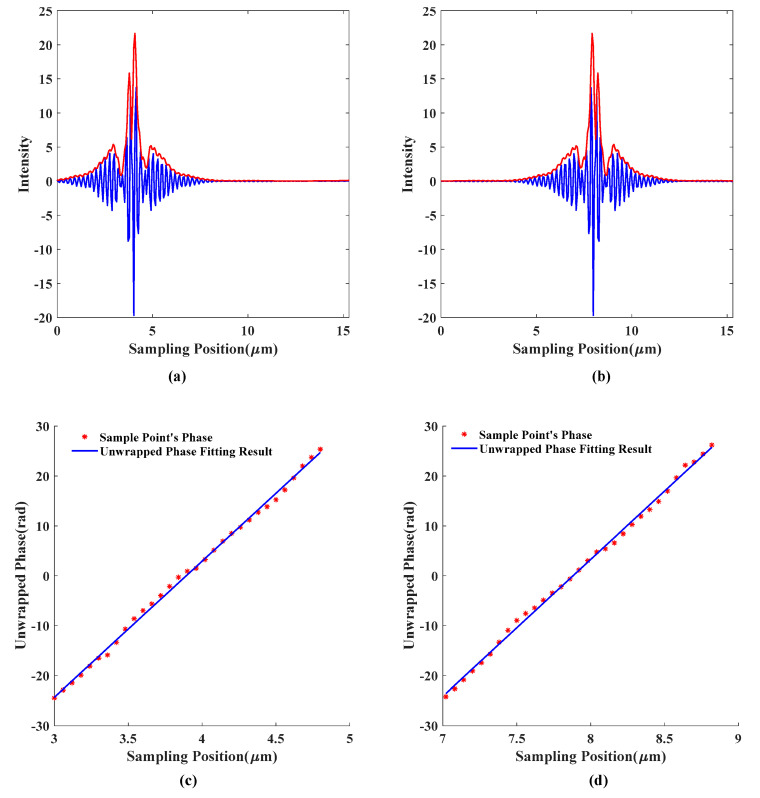
The calculation results of the proposed algorithm: (**a**) the solution effect of the *z*_0_ = 4 μm simulation interference signal and signal envelope; (**b**) the solution effect of the *z*_1_ = 8 μm simulation interference signal and signal envelope; (**c**) the signal phase information is obtained by the *z*_0_ = 4 μm solution; (**d**) the signal phase information is obtained by the *z*_1_ = 8 μm solution.

Figure 11 shows the information obtained in the solution process of the algorithm proposed in this paper. Figure 11a,b show the simulated interference signal and signal envelope at the step-down surface *z*_0_ = 4 μm and the step upper surface *z*_1_ = 8 μm, respectively. Figure 11c,d show the phase information of the signal obtained by the solution, take the maximum position of the envelope peak as the reference zero point to carry out phase unwrapping, and the phase of unwrapping is straight-line fitted by the least square method.

Table 2 compares different algorithms to calculate the simulation data of standard step with 10 times. It can be seen from the results that due to the large intensity change near the zero-order fringes of the white LED interference signal, it has obvious inferior influence on the reconstruction accuracy of the WLPSI algorithm, and the repeatability of the solution is the worst. The centroid method and the FFT method are also affected by the light intensity fluctuation near the zero-order fringe, but the repeatability of the calculation results is better than that of WLPSI. The accuracy and repeatability of the algorithm proposed in this paper are the best. Simulation experiment proves the reliability of the algorithm.

## 5. Experiments and Results Discussion

In order to verify the simulation results of the proposed algorithm, the LED WLSI experimental system is built, and selects the CREE brand warm white LED (2600k–3700k illuminance) as the lighting source. The PZT is equipped with an interference objective lens, which is controlled by a computer and moves in equal steps in the *Z* direction at specific intervals. Every time the PZT takes a step, the CCD records an interferogram and stores it in the computer. Finally, the collected set of interferograms is solved to obtain the 3D contour of the measured object.

### 5.1. Standard Step Sample Measurement

**Figure 12:**
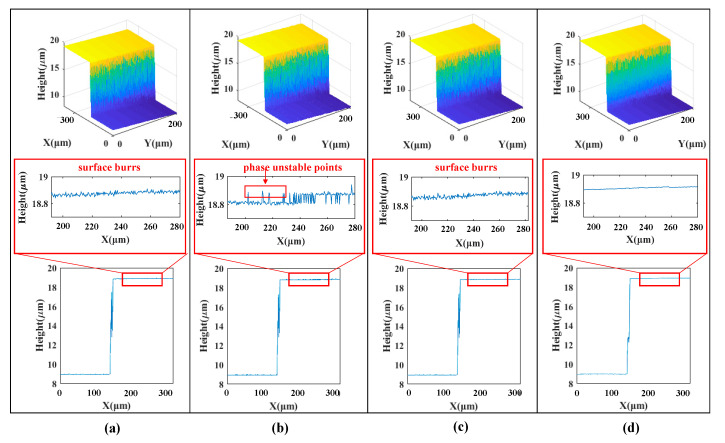
Reconstruction results of a standard step measurement: (**a**) Centroid; (**b**) FFT; (**c**) Stoilov WLPSI; (**d**) Proposed Method.

The system repeats 10 measurements on a standard step with a nominal height of 9.976 ± 0.0028 μm. In the measurement process, there is a phenomenon that the interference fringes on the upper and lower surfaces of the step are unevenly distributed due to the dual wavelength characteristic of the LED spectrum. The centroid method, the FFT envelope method, the Stoilov five-step phase shift method and the algorithm proposed in this paper are used to solve the standard step interferograms. The 3D shape of the step and the two-dimensional contour at the same cross-section obtained by different algorithms are shown in Figure 12. Table 3 compares the measurement results of 10 sets of the step height with the four algorithms. It can be seen from Figure 12 that the algorithm proposed in this paper has the best surface quality reconstruction, and there is no slight burr. At the same time, the accuracy and repeatability of the step height measurement results are better than the Centroid, FFT and Stoilov WLPSI algorithms. The results of the measurement experiments accord well with the simulation experiments, which proves the reliability of the algorithm proposed in this paper.

### 5.2. Continuous Surface Measurement Experiment

**Figure 13:**
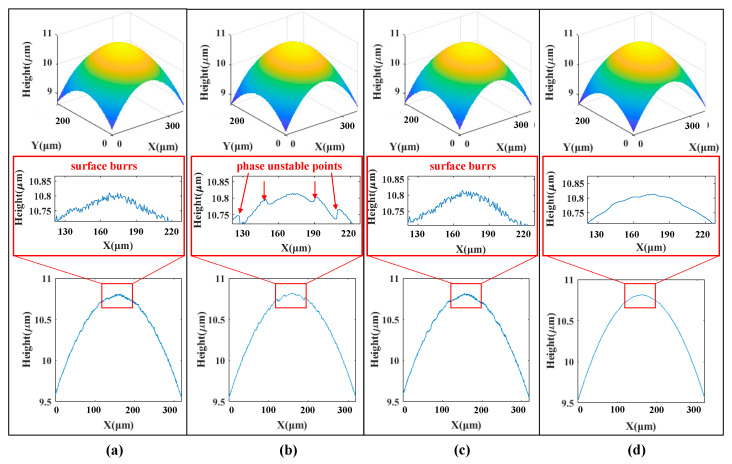
Reconstruction results of standard sphere measurement: (**a**) Centroid; (**b**) WLPSI; (**c**) FFT; (**d**) Proposed algorithm.

Measurement experiments on continuous curved surface and optical freeform elements are carried out to further verify the superiority of the proposed algorithm. The standard ball and hexagon compound eye structure processed by ultra-precision lathe were measured. The surface of the two components is smooth and the surface quality is very high. The reconstruction results of the three algorithms are compared with those of the proposed algorithm. The unevenness of the interferogram fringes caused by the influence of the LED itself also appears on the spherical and hexagon compound eye arrays. The measurement results of the spherical surface are shown in Figure 13. The two-dimensional contour of the same cross-sectional position is shown in Figure 13a–c below. It can be seen that the reconstructed surfaces all show strong noise. However, the calculation results of the algorithm proposed in this paper are shown in Figure 13d below, with better smooth continuous contour. 

**Figure 14:**
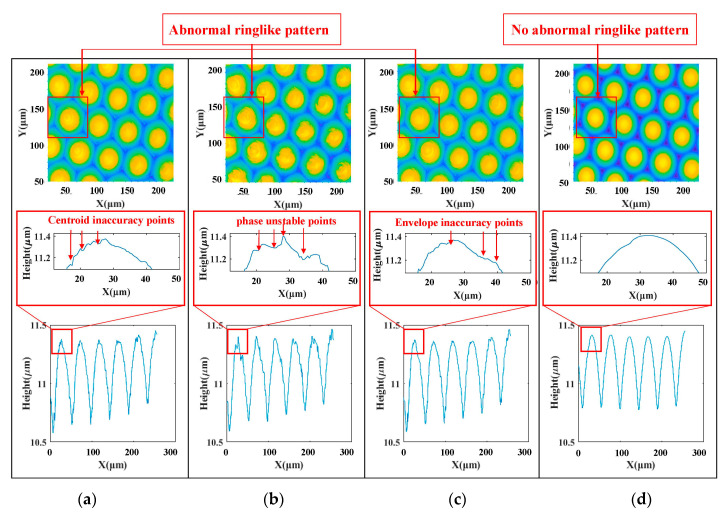
Reconstruction results of hexagonal compound eye array measurement: (**a**) Centroid; (**b**) WLPSI; (**c**) FFT; (**d**) Proposed algorithm.

The measurement results of hexagon compound eye arrays are shown in Figure 14. As shown in Figure 14a–c below, the two-dimensional profile at the same cross-section position is not ideal. There are obvious rough points in the measurement results of the profile, the surface profile is not smooth and there are surface fluctuations similar to the abnormal ringlike pattern. The effect of the proposed algorithm is shown in the Figure 14d below, it can be seen that the algorithm proposed in this paper can effectively improve the quality of surface measurement, and solves the problems of surface burrs, residual abnormal ringlike pattern and phase unstable points on reconstructed surface, which cannot be accomplished by traditional white light interference algorithms. The algorithm proposed is not affected by the uneven contrast of the interferogram caused by the white LED, it can effectively realize the surface reconstruction of VSI measurement data of continuous surface or freeform surface periodic structure under LED light source, which proves that the algorithm has high feasibility.

## 6. Conclusions

In this paper, aiming at the problem of the interference measurement caused by the peak’s spectrum characteristics of a white LED light source, the influence of white LED light source on the VSI is analyzed by modeling and simulation. A new surface recovery algorithm is proposed and the algorithm is mathematically derived. The new algorithm has strong anti-interference ability and can effectively solve the problem caused by the dual-wavelength spectrum in the LED WLSI and realize the surface reconstruction of VSI measurement data of freeform surface under the white LED. For the measurement of 10 μm standard step height, the accuracy of the solution is compared by three traditional algorithms. The experimental results show that the new algorithm has good repeatability and stability. In addition, the spherical and hexagonal compound eye array measurements experiments show that the algorithm solves the problems of surface burrs, residual abnormal ringlike patterns and phase unstable points on the reconstructed surface, and it has a good smooth measurement effect in continuous surface profile measurement, proving that the new algorithm has high feasibility.

## Figures and Tables

**Table 1:** Zero-order fringe positioning accuracy of simulated white light interference signals under different envelope algorithms.

	Mean Deviation Using White LED (μm)	Standard Deviation Using White LED (μm)	Mean Deviation Using Halogen Lamp (μm)	Standard Deviation Using Halogen Lamp (μm)
Centroid	0.0069	0.0036	0.0019	0.0009
FFT	0.0072	0.0032	0.0018	0.0016
Stoilov WLPSI	0.0236	0.0183	0.0143	0.0162

**Table 2:** Comparison simulation results for 4μm step heights using the four algorithms.

	Centroid	FFT	Stoilov WLPSI	Proposed Method
Average Step Height (μm)	3.985	3.987	3.986	3.994
Standard deviation (μm)	0.0126	0.0081	0.0176	0.0045

**Table 3:** Reconstruction results of different algorithms for measuring 9.976 ± 0.028 μm standard steps.

Methods	Centroid	FFT	Stoilov WLPSI	Proposed Method
Average Step Height (μm)	9.959	9.960	9.953	9.967
Standard deviation (μm)	0.0187	0.0172	0.0400	0.0041
